# Cocoa Mucilage as a Novel Ingredient in Innovative Kombucha Fermentation

**DOI:** 10.3390/foods13111636

**Published:** 2024-05-24

**Authors:** Rossy Rodríguez-Castro, Raquel Guerrero, Antonio Valero, John Franco-Rodriguez, Guiomar Posada-Izquierdo

**Affiliations:** 1 Facultad de Ciencias de Industria y Producción, Universidad Técnica Estatal de Quevedo, Quevedo 120301, Ecuador; rlrodriguez@uteq.edu.ec (R.R.-C.); rguerrero@uteq.edu.ec (R.G.); 2 Department of Food Science and Technology, UIC Zoonosis y Enfermedades Emergentes ENZOEM, CeiA3, Universidad de Córdoba, 14014 Córdoba, Spain; bt2vadia@uco.es; 3 Carrera de Agropecuaria, Facultad de Educación Técnica Para el Desarrollo, Universidad Católica de Santiago de Guayaquil, Guayaquil 09014671, Ecuador; john.franco@cu.ucsg.edu.ec

**Keywords:** *Theobroma cacao* L., secondary components (mucilage), fermented drink, high nutritional performance, use, valorization

## Abstract

Cocoa tree plantations aim to harvest grains found in the cob to produce cocoa and chocolate. There has been a growing interest in valorizing the secondary components of the cocoa fruit, such as the peel, placenta, and mucilage/pulp, as valuable sources of nutrients for healthy food preparation. In other words, by-products derived from these raw materials are an exploitable source of nutrients in the preparation of healthy food. In the present study, two varieties of cocoa, *National Cocoa Fino de Aroma* (NCFA) and *Colección Castro Naranjal 51* (CCN-51), were evaluated and harvested during both dry and rainy seasons. This evaluation was based on the profiling of the cob, peel, grain, placenta, and mucilage in different stages of ripeness (underripe, ripe, and overripe). Also, from the ripe raw material, a fermented beverage prototype was developed, such as kombucha, with different concentrations of mucilage (40, 60, 80, and 100 g/L). Physicochemical analyses, such as acidity, °Brix, pH, moisture, ash, protein, fat, fiber, vitamins, sugars, and polyphenols of the raw mucilage material and acidity, °Brix, and pH values of the fermented kombucha, were carried out. The best performances were obtained with the CCN-51 variety in the rainy season. Among the fermented drink panelists, the CN40 treatment (Nacional Mucilage + 40 g/L of sugar) received the highest acceptability and was considered the best. Given its efficiency, nutritional content, and potential applications, this product presents a promising strategy to address Sustainable Development Goals related to zero hunger, health and well-being, and climate action.

## 1. Introduction

The cocoa tree, also called *Theobroma cacao* L., has been known since ancient times. The harvest of cocoa comes from the American continent, although the area of provenance is not accurately known. Various investigations hold that its beginning was generated from the Toltec culture due to a Quetzalcóalt belief that credited the tree to have multiple healing virtues [1]. From there, the knowledge and use of this tree expanded to other cultures, such as the Olmecas, Aztecs, and Incas [2]. The productivity of the cocoa tree in convenient conditions starts at three years of age and can give cobs all year, depending on the area of production and altitude. From 5 years of age, the production is considered fulfilled, and its life expectancy is 100 years of age. However, the optimum time of productivity is around 40 years. The cocoa cob is a suitable ingredient to be used throughout the year. Nonetheless, during the stages of its development, farming can undergo various alterations that affect the production of cobs, such as phytosanitary problems, moniliasis, Black Pod, and Witch’s broom, which can cause up to 60% loss of production. All these changes are mainly produced by the effects of the climate [3].

The cultivation of cocoa is predominantly practiced on a large scale in regions characterized by humid and hot climates in countries such as Ecuador, Colombia, Venezuela, Brazil, Peru, Bolivia, Trinidad and Tobago, and Mexico. It serves as a major source of income for agricultural families [4].

Ecuadorian cocoa stands out for its global exportation to around 61 countries, making a significant economic impact on households [5]. Since 2021, the total area of cocoa plantations in Ecuador was to an average of 626.962 hectares, representing 26% of the country’s total agricultural area [6]. Within these territories, diverse varieties of cocoa are cultivated. Notably, *National Cocoa Fino de Aroma* (NCFA) covers 25% of the planted area, while the remaining 75% belongs to *Colección Castro Naranjal 51* (CCN-51) [7]. Regarding the data registered in the Plant and Animal Protection Control and Regulation Agency, cocoa is exported in different formats: vegetable product and as a by-product of vegetable origin, creating other products such as oil, blocks, bars, peel, husk and film, chocolate, and other prepared foods that contain cocoa [8,9]. NCFA is appraised for its fruity, floral, herbal, and woody flavors, standing out from other varieties and being used to create intense and balanced chocolates. Its origin and domestication are in the Amazon region of the south of Ecuador [8]. On the other hand, the CCN-51 variety, a cloned cocoa of Ecuadorian origin, was declared to be of high productivity in 2005, being valued for its high tolerance to diseases, efficiency, and quality [10].

In Ecuador, only 20–23% of the total cocoa production is used in the final product, indicating that the cocoa beans are exclusively utilized [11]. Between 77 to 80% of the remaining by-product is discarded, including the mucilage/pulp. The by-products derived from these raw products mean a significant source of nutrients that could be used to elaborate healthy foods, thus contributing to diversifying the range of foods presented to the population [12].

The average nutritional composition of cocoa consists of proteins (11.5%), starch (7.5%), tannins (6%), water (5%), salts and trace elements (2.6%), organic acids (2%), theobromine (1.2%), and caffeine (0.2%), among others [13]. However, the nutritional composition of the by-products of cocoa processing is not well studied because they are considered for use.

At an industrial level, the peel, placenta, and mucilage are often discarded. However, utilizing these resources to create other products introduces a new perspective on sustainability, which helps reduce environmental problems and creates additional sources of income [14].

In recent years, by-products derived from cocoa mucilage have begun to be commercialized, although their usage is still relatively limited. Their adoption has been facilitated by their fruity flavor and natural sweetness, requiring no additional sugars. On the other hand, mucilage has been pasteurized to preserve its freshness, and later used as a natural sweetener in bars or in chocolate covers, besides being transformed into lyophilized powder to be sold in its most natural form [15]. In the same context (its use), artisanal chocolate-makers and now also more famous brands are experimenting with including mucilage in their chocolate bars, taking it always as a more natural and healthier sweetener. It is interesting to note that cocoa mucilage exhibits various flavor profiles depending on its variety and origin, similar to cocoa beans [16].

Ref. [17] defines mucilage as a substance covering the seeds of cocoa, traditionally used as substrate in the fermentation process of the grains of cocoa, and is also an important element in the formation of precursor substances of scent and flavor. This by-product of cocoa production holds a high nutritional and functional value, due to its content of vitamins B, C, D, and E and minerals such as Ca, Fe, K, Mg, and Zn [18]. Mucilage is capable of “metabolizing” during the fermentation of the bean of cocoa as it contains bacteria and yeasts that play a crucial role in the metabolism of sugars present in the mucilage. These microorganisms participate in the production of compounds that positively affect the organoleptic characteristics of cocoa, contributing to the unique flavor profile of the chocolate. Changes in the chemical composition of the mucilage layer can affect the production of acetic acid by yeasts and bacteria, and the consequences of these interactions are multiple.

Firstly, the organoleptic quality of the cocoa, including its flavor and scent, may be affected. In addition, changes in the production of acetic acid may influence the texture and acidity of the fermented cocoa, which has a direct impact on the final quality of the chocolate from these fermented beans of cocoa [19].

The utilization of cocoa mucilage has gained significant importance in recent years, as evidenced by various studies. These studies have led to the development of different derived products, including non-alcoholic and alcoholic beverages, wines, vinegars, jellies, ice creams, gels, and marmalades [15,20]. Additionally, cocoa mucilage shows great potential in pectin production, making it an ideal substance for achieving consistency in marmalades and jellies [21]. The emergence of cocoa mucilage as a valuable market product provides cocoa producers with a new source of income.

Previous investigations emphasize that mucilage should not be regarded as a mere by-product of cocoa, as it possesses chemical properties of significant value and a distinctive sensory profile [22,23]. Its tropical, fresh, and fruity characteristics resemble a blend of fruit juice. Some companies elaborate cocoa drinks exclusively from this mucilage (or white pulp), without the need for added sugars [24,25].

**Figure 1:**
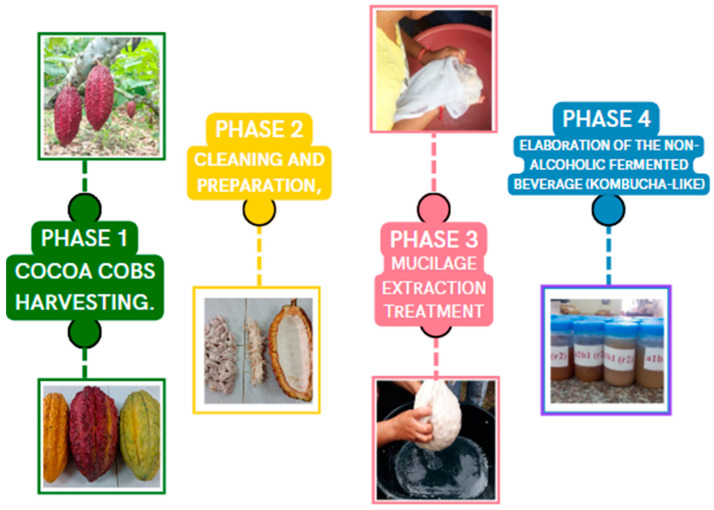
Description of the process of extraction of the mucilage of cocoa.

**Figure 2:**
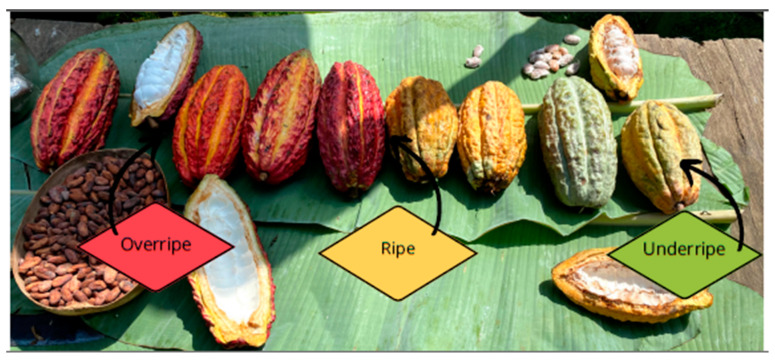
Cocoa cobs at different stages of ripeness at the time of harvest, from overripe (left) to underripe (right).

The aim of this study lies, firstly, in an evaluation of the pondered performance of the two varieties of cocoa, *Theobroma cacao* L. (*National Cocoa Fino de Aroma* (NCFA) and *Colección Castro Naranjal 51* (CCN-51)), harvested during the dry and rainy seasons. Such evaluation will be carried out regarding the different constituent parts of cocoa: cob, peel, grain, placenta, and mucilage, with special emphasis on the maturation stages (Figure 1 and Figure 2: underripe, ripe, and overripe). Secondly, a novel procedure will be implemented to extract overripe mucilage, with the aim of producing a fermented non-alcoholic beverage (kombucha) and conducting sensory evaluations for various formulations.

## 2. Materials and Methods

### 2.1. Research Design

This investigation took place in the Experimental Campus “La María” of the Technical State University of Quevedo (UTEQ), Los Rios Province, Ecuador. In Figure 1, the different phases of the elaboration process are represented. The experimental period covered two consecutive years, where harvesting periods occurred in May, June, and July (dry months), as well as in November, December, and January (rainy months). The dry season in Quevedo generally spans from June to November. During this period, temperatures are warm during the day and can vary depending on the specific month, ranging between 22 °C and 32 °C. In contrast to the rainy season, Quevedo experiences more hours of sunlight during the dry season, averaging 7 to 9 h of sunlight per day. This increased sun exposure contributes to a drier and sunnier climate in the region. Additionally, humidity levels are lower during the dry season, fluctuating between 60% and 80%. Although humidity is lower than during the rainy season, it remains relatively high due to the tropical climate of the region. During the rainy season, which typically occurs between January and May, Quevedo experiences a significant increase in precipitation. The amount of rainfall tends to be higher, averaging between 1500 to 2000 mm per year. This can lead to temporary flooding and river overflow, especially in areas near bodies of water. Temperatures during the rainy season remained warm, with lows rarely dropping below 20 °C and highs that can exceed 30 °C. The combination of high temperatures and humidity during the rainy season can contribute to a feeling of mugginess in the region, with humidity levels typically ranging between 80% and 90%.

Phase 1. Fruit harvesting: Cocoa cobs were recollected in two specific moments of years, 2019 and 2020. For this study, two types were considered: *National Cocoa Fino de Aroma* (NCFA) and *Colección Castro Naranjal 51* (CCN-51). One thousand eight hundred cobs were gathered in total. At the same time, the harvesting periods were April, May, and June (dry months) and October, November, and December (rainy months) in both years of study. Harvesting was conducted in the morning, selecting cobs at various stages of maturity, with each cob weighing approximately 300 g. After harvesting, they were transported to the processing plant at room temperature in fruit collection baskets, where Phase 2 took place.

Phase 2. Cleaning, preparation, and process of extraction of mucilage and other components of the cocoa cob: Cobs were put under cleaning and sanitizing treatment with tap water and sorted out according to the variety, time of the year, and maturation state. Maturation state is identified according to the color of the external part of the cob. In NCFA, the unripe ones are green, whereas the ripe ones are yellow. In the case of CCN-51, they are red and orangey yellow, respectively. In the case of overripe cobs, both varieties show a brown color.

After being washed, cobs were transported to proceed with the extraction of the grains, placenta, and mucilage with stainless steel machetes. The extraction of the components was performed using two longitudinal and two transversal cuts, which allowed the opening of the cob. In this process, the weight of the full cob, the peel, the bean, and the mucilage were measured. After writing down the weight of all the components, they were kept at refrigeration (20 °C) to later be pasteurized and fermented. Two liters were poured into the traditional glass wine bottles, and each bottle was sealed with a cork stopper.

Phase 3. Mucilage extraction treatment: The treatment followed the artisanal process, which involves wrapping the cocoa bean in a thin cloth and applying manual pressure to extract the mucilaginous liquid surrounding the cocoa bean. It should be emphasized that the extraction process was not taken to the final stage, as the cocoa bean requires a certain amount of mucilage to complete the fermentation process and acquire the essential characteristics necessary for chocolate production. Using this method, the extraction efficiency of the mucilage increases up to 80%.

After being washed with tap water, the pods were transported to proceed with the extraction of the beans, placenta, and mucilage, respectively. Stainless steel machetes were used to make cuts in the pod, performing two longitudinal and two transverse cuts to fully open the pod. During this process, the weight of the complete pod and its components, including the husk, the kernel, and the mucilage, was recorded. Subsequently, they were kept refrigerated at a temperature of approximately 20 °C.

In Phase 3, corresponding to the mucilage extraction treatment, an artisanal method was employed. This involved wrapping the cocoa bean in a fine linen cloth and manually applying pressure to the bean (graphical abstract). This way, the liquid surrounding the cocoa bean, known as mucilage, was obtained and collected in aluminum trays for later use. It is important to note that the extraction process was not carried out in its entirety, as the cocoa bean requires a certain amount of mucilage to remain integrated into its shell to complete the fermentation process and acquire the essential characteristics necessary for chocolate production. By using this method, the mucilage extraction efficiency increases by up to 80%.

In Phase 4: Elaboration of the non-alcoholic fermented beverage (kombucha-like): The experiment began (Figure 1 in Phase 3) with ripe stage mucilage and involved eight treatment combinations (see Table 1). The ingredients used were: 2 L of cocoa mucilage, 10 g of green tea, market yeast (120 mL of starting ground), and sucrose as a source of added sugar (regarding the treatments in Figure 1). Factorial design A × B with two levels in Factor A (NCFA and CCN-51) and four levels in Factor B (40, 60, 80, 100 g/L). For this, 8 treatments with 4 repetitions each and a total of 32 samples of fermented beverages were evaluated.

### 2.2. Activities Involved in the Process of Elaboration of the Fermented Beverage

**Figure 3:**
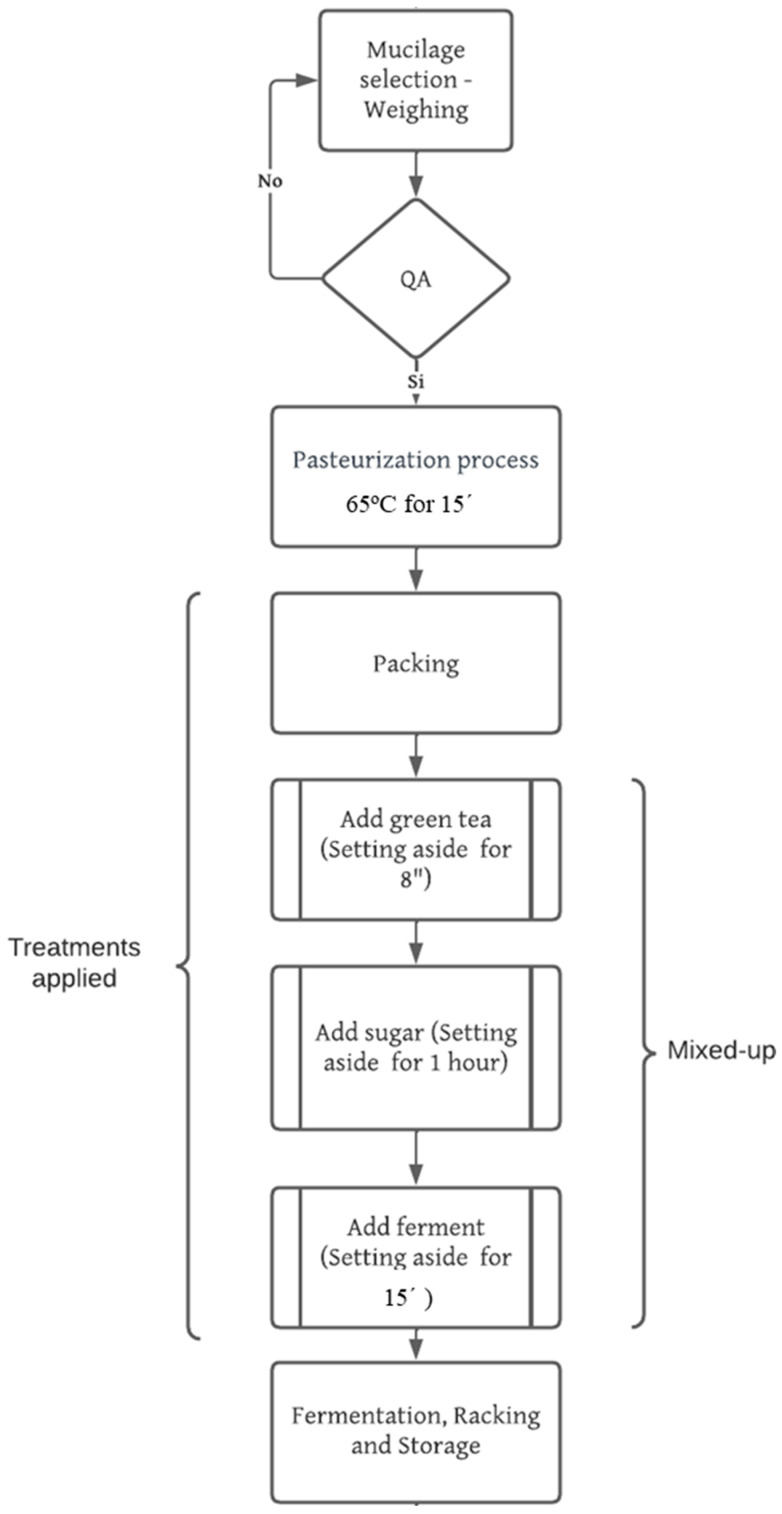
Flow chart of the elaboration process for the fermented non-alcoholic cocoa mucilage beverage.

It started with the pasteurization of fresh cocoa mucilage in a metallic container, subjected to a temperature of 65 °C for 15 min (Figure 3). Subsequently, tea preparation ensues, during which 10 g of green tea is added to a volume of 2 L of pasteurized mucilage. This mixture was heated for the following 8 min until it acquired a slightly dark color, aiding in preventing light filtration that may interrupt the fermentation process, resulting in a highly concentrated solution. Following this, sugar was added according to the designated treatment combination (see Table 1) in the various treatments. To obtain the fermented beverage, the mucilage was left for approximately one hour until both the tea and mucilage reached an ambient temperature of 28 °C, at which point the mixing process was undertaken. Careful integration of both solutions was conducted in the same container, followed by gentle agitation to ensure proper homogenization. Subsequently, the liquid was poured into a glass container containing the SCOBY (Symbiotic Culture Of Bacteria and Yeast), ensuring complete coverage, and then tightly sealed with filter paper or absorbent towels, allowing for slight ventilation to facilitate gas exchange necessary during the fermentation process. The container was stored in a dark, dry place for 15 days and housed in a previously sterilized glass jar at a constant temperature of 25 °C. After this period, the product was packaged through a filtration process and placed in sterilized glass bottles. Final storage occurs under refrigeration to slow down or halt fermentation, ensuring product stability. Finally, the resulting beverage underwent physicochemical and sensorial analyses.

### 2.3. Physicochemical Analysis

For the physicochemical parameters (such as acidity, °Brix, pH, moisture, ash, protein, fat, fiber, vitamins, sugars, and polyphenols), cocoa mucilage samples of the CNFA and CCN51 varieties were used in a ripe state (as a raw material for the fermented beverage). Finally, the acidity, °Brix, and pH values were determined in the 8 formulations studied in the production of the beverage. All methodologies followed the AOAC for *Physicochemical Analysis in food.*

### 2.4. Sensorial Analysis

For the sensory analysis, a panel of experts comprising 30 semi-trained panelists participated. The beverage was analyzed after being elaborated for 16 days. In the hedonic test, the following attributes were evaluated: color, flavor, texture, smell, sweetness, and aftertaste. Each tester evaluated 8 samples. The sensory analysis report utilized the following scale: strongly dislike (1), dislike (2), neutral (3), like (4), and love (5).

### 2.5. Statistical Analysis

To compare the average numbers of the treatments, the Tukey multiple range test was used (*p* < 0.05), and Kruskal–Wallis (*p* < 0.05) non-parametric analysis was used for the sensorial results; both with the statistics software (InfoStat, 2017).

## 3. Results and Discussion

### 3.1. Characterization of the Cocoa Cobs

In Table 2, the results obtained regarding the variety of cocoa at different moments of the year are observed. The average weights of the whole cob of cocoa show that the rainy season gave bigger values in the overripe sample, corresponding to the CCN-51 variety (804.55 g). On the contrary, the lowest weight (281.86 g) was observed in the same variety but in the dry season and with the fresh underripe sample. The disagreement between the weights of the cocoa cobs depending on the variety was also noticeable. In this case, it is noted that the CCN-51 variety produced heavier cobs during the rainy season compared to the dry season.

In Table 3, the average weights of the different components of the cocoa cob are shown, evidencing that from both varieties, more amount of mucilage was obtained in the rainy and in the overripe state of maturation (123.44 g and 160.91 g for the NCFA and CCN-51 varieties, respectively). The fermented beverage was prepared using ripe mucilage to achieve better performance and increase the quantity of mucilage obtained.

**Figure 4:**
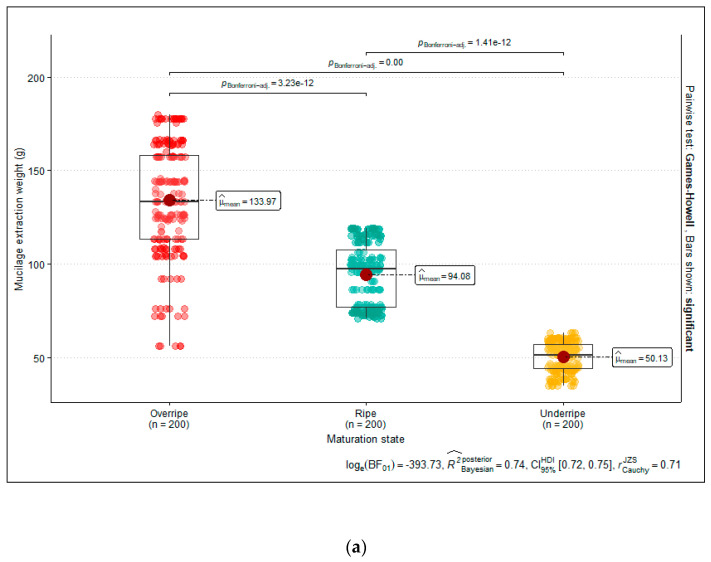

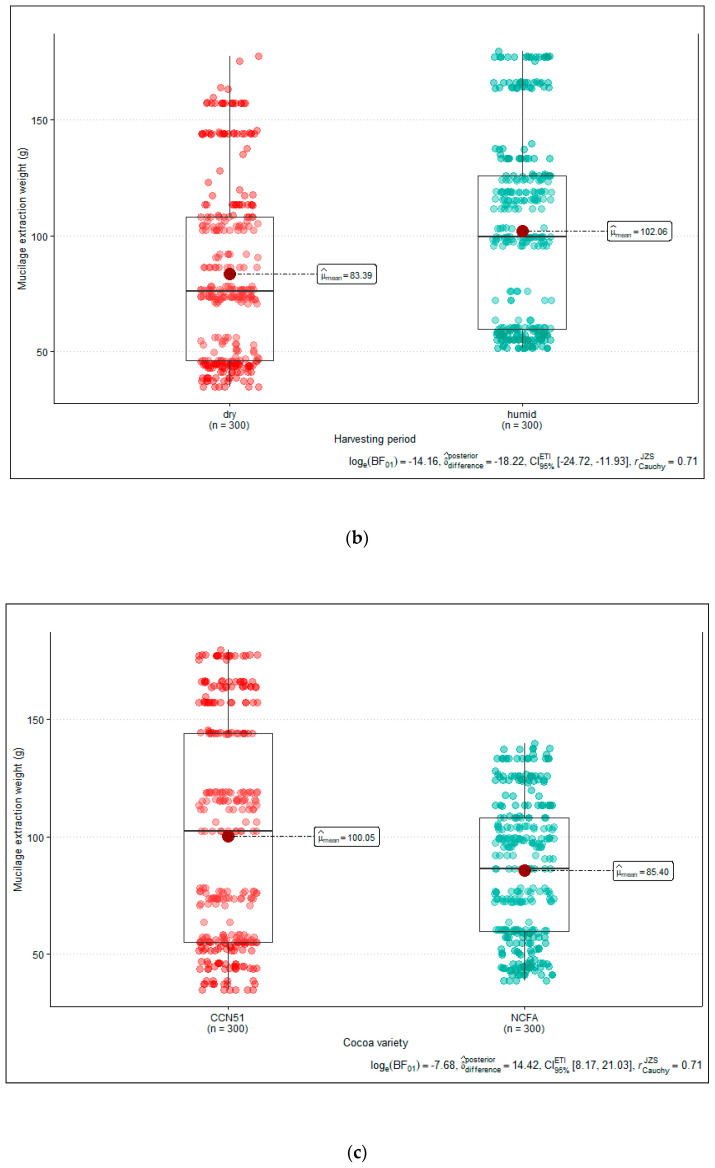
(**a**) Boxplots of the individual mucilage extraction weights depending on the maturation state of the fruit when harvested. (**b**) Boxplots of the individual mucilage extraction weights depending on the harvesting season. (**c**) Boxplots of the individual mucilage extraction weights depending on the cocoa variety.

Figure 4a–c show the individual average weights for mucilage extraction according to the variety, season, and maturation state of the cocoa cobs. Neither the cocoa variety nor the season affected the extraction efficiency (*p* > 0.05), though there was high variability among the tested samples. However, the maturation state influenced the extraction efficiency, finding significant differences between overripe, ripe, and underripe cocoa cobs. An increased data dispersion was obtained for the overripe samples (Figure 4a).

To ensure compliance with the necessary physicochemical and nutritional parameters (such as acidity, °Brix, pH, moisture, ash, protein, fat, fiber, vitamins, sugars, and polyphenols), cocoa mucilage samples of the CNFA and CCN51 varieties were used. This was carried out to assess suitability as a raw material for kombucha production.

In Table 4, the acidity values recorded for the CNFA variety were 1.35%, and for the CCN51 variety, 1.44%. These results are consistent with the findings reported by [26], who pointed out that cocoa pulp typically has an acidity range between 0.77% and 1.62%.

In the study carried out by [27], cocoa products were examined, finding that this product contained 60.54% (dry matter-based) dietary fiber, predominantly composed of insoluble fiber, although with significant amounts of soluble dietary fiber (10.09% dry matter basis). In contrast, the fiber values recorded for the CNFA variety were 8.56%, and for the CCN51 variety, 8.35%.

The results obtained in this study for the polyphenol variable show lower values than those reported by Martínez et al. [28]. These authors found a total polyphenol content in cocoa mucilage samples from Cono and Taura in the province of Guayas, extracted in a methanol–water solution, ranging from 173.67 to 182.63 mg AGE•100 mL^−1^. In contrast, the phenolic content of the extracted samples containing the CNFA variety was 107.03 mg, and for the CCN51 variety, 67.43 mg.

### 3.2. Fermented Beverage

**Figure 5:**
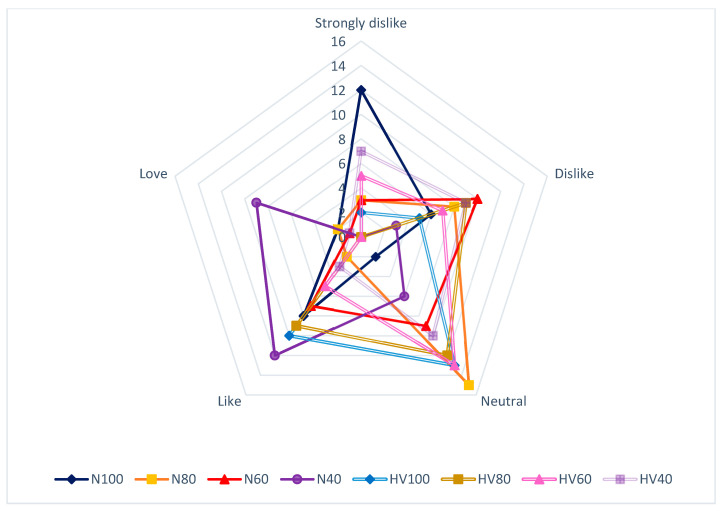
Results of the organoleptic analysis of parameter flavor in the fermented drink.

In the sensorial analysis performed by the panelists, the treatment of NCFA 40 stands out as the sample with the best rating in all characteristics apart from aftertaste, where it was described as indifferent and, thus, irrelevant (Table 5). The sensorial attribute of the fermented beverage identified by the panelists as the most important was flavor (Figure 5), with N40 being the best punctuation, with a score of 4.84/5 from 70% of testers. Between the rest of the treatments, there was a significant difference. On the contrary, the treatment HV100 (Hybrid Variety Colección Castro Naranjal 51 (CCN-51) + 100 g/L sugar) and N100 (National Cocoa Fino de Aroma (NCFA) + 100 g/L sugar) presented a score of 2.92/5, given by 40 and 43% of testers, respectively.

While it is true that this fermented beverage with cocoa mucilage has never been elaborated before, and therefore, there are no comparative studies available, there is an intention to assess its performance based on results obtained from similar products in other food matrices. For instance, a study by [29] evaluated a fermented drink based on lemon (*Citrus limon*), where flavor was the most important attribute, similar to our study. In their study, they reported a score of 4.25 out of 5 for flavor, indicating a “very good” result, which serves as a reference for acceptability among panelists.

For the attribute smell, sample N40 obtained the highest mark (4.79/5) yet the sample given the lowest mark was HV100 with 3.25/5, evidencing the fact that the smell has no significant difference among treatments, given that most scores for all the samples are “dislike” or “neutral”, apart from HV40, where 50% of the testers identified the smell to be strongly dislikeable. These values can be better observed in the Supplementary Material in Annex S3.

In fermented beverages, the attribute of smell is important, as demonstrated by [30]. They conducted a study elaborating and characterizing a fermented drink, such as kombucha, using bamboo leaves and mulberry tree leaves. In their findings, they noted that while the color and flavor of the kombucha made from bamboo leaves were not significant, they were not useful attributes for evaluating the formulations. As a result, more relevance was given to the attribute of smell.

In our study, the attribute of color was found to be not relevant, as it showed no significant differences, and testers rated it as “like”. However, the best score was obtained for N40, maintaining its trend across the rest of the attributes. In contrast, in other studies such as that by [31], the color attribute was very important, and also in [32], evaluating six different formulations of Zijuan tea-based kombucha, significant differences in color were observed. In their study, the highest rating was obtained for the salmon pink color, which was uncommon among tea drinks.

**Figure 6:**
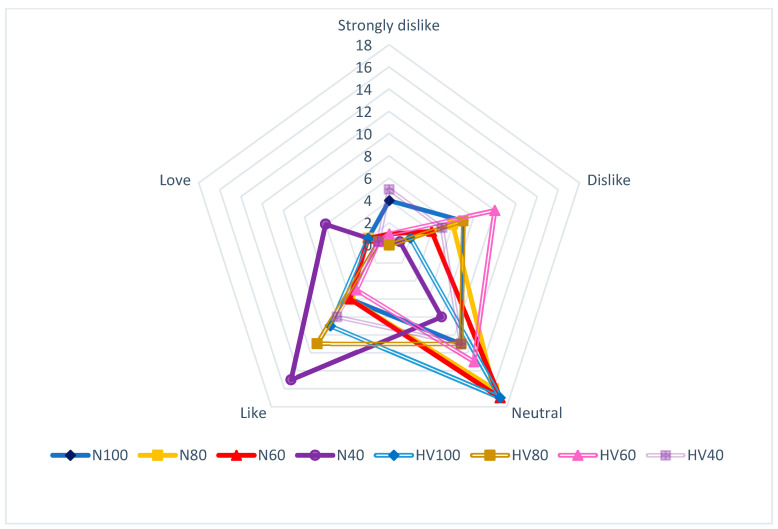
Results of the organoleptic analysis of the texture parameter of the fermented drink.

In Figure 6, we observe the texture parameter, where N40 scored the highest rating among 70% of the panelists. Conversely, the texture of HV60 was disliked by 33% of the panelists. While most samples exhibited an indifferent perception of this attribute, there were significant differences in the ratings (*p* < 0.05). This finding is consistent with the results of [33], who conducted sensory tests on texture using a nine-point hedonic scale. Their fermented beverage obtained a score of 7/9 (“medium like”), demonstrating that the kombucha beverage elaborated in the present study aligns with the expected results for this type of product.

Finally, the aftertaste attribute in the cocoa mucilage fermented beverage was less relevant for the panelists, who valued it as indifferent. On the other hand, sweetness obtained low marks and with a bigger significant difference. This stands within the expected results, given that it is a key factor in the different studied formulations. N40 treatment was “liked” by 66% of panelists, showing similar behavior to the observed in the study developed by [34], where they assessed the fermentation in fine coco type Scavina; and [35] in Kombucha based on vine tea and sweet tea.

In Table 6, a significant difference in the pH of the fermented beverage was observed (*p* < 0.05), with a notable variation between samples with the highest (3.87, NCFA40) and lowest (2.20, NCFA100) pH levels. Regarding the °Brix content, the highest value was recorded at 6.12 (NCFA40), while the lowest was 12.46 (NCFA100). In terms of acidity, the highest value was found at 0.72 (CCN100), while the lowest value was 0.17 (CCN40). All these results are within what is expected for a non-alcoholic fermented beverage [36]. Therefore, studies show that the chemical composition of the kombucha is directly linked to the ingredients and their proportions, as well as the variation of fermentation parameters. Thus, these variations can enhance the production of specific nutritional compounds [37].

Finally, cocoa mucilage is a novel ingredient in innovative kombucha fermentation. Given its efficiency, nutritional content, and potential applications, this product presents a promising strategy to address Sustainable Development Goals related to zero hunger, health and well-being, and climate action, as the data displayed by [18], where bioactive compounds and beneficial health effects were studied. The use of mucilage, with its high performance and significant nutritional value, positions it as a promising strategy to address the SDOs in underdeveloped countries since it contributes to zero hunger, health, well-being, and climate action.

## 4. Conclusions

The varieties considered for this study were: Cacao Nacional Fino de Aroma y Colección Castro Naranjal 51 CCN-51. In total, 1800 cobs were recollected and grouped by variety, season of the year, and state of maturation, with the best state to obtain mucilage being the overripe stage, with over double performance than in unripe.

The performance based on the weight of the cobs obtained better results for the ripe varieties in the rainy season compared to dry, although the variability of the samples was higher in this last season for all the stages of maturation of both varieties of cocoa, apart from the National Cocoa in the winter season, which shows a range of weights from 430 to 600 g per cob and SD of 89.92.

The non-alcoholic fermented beverage (kombucha) received an excellent score, with the N40 (national + 40 g/L sugar) being the best combination in terms of flavor, texture, and sweetness. Aftertaste was not relevant to any of the panelists (the higher score was “neutral”), contrary to flavor and texture, which were determined in order to choose the best treatment and, finally, the acceptance of the new non-alcoholic fermented beverage based on mucilage and green tea.

The use of mucilage, with its high performance and significant nutritional value, positions it as a promising strategy to address the SDOs in underdeveloped countries since it contributes to zero hunger, health, well-being, and climate action.

## Figures and Tables

**Table 1:** Treatments used in the production of kombucha (eight treatment combinations with ripe stage mucilage).

Treatment	Sample	Sugar Concentration (g/L)
T1	N100	100
T2	N80	80
T3	N60	60
T4	N40	40
T5	HV100	100
T6	HV80	80
T7	HV60	60
T8	HV40	40

N = National Cocoa Fino de Aroma (NCFA); HV: hybrid Variety Colección Castro Naranjal 51 (CCN-51).

**Table 2:** Average weights of cob with regard to the season of recollection and maturation in *n* = 150 units.

		Dry Season			Rainy Season		
Variety	Maturation	Weights Rank (g)	x̅ Weights (g)	SD	Weights Rank (g)	x̅ Weights (g)	SD
NCFA	Underripe	240–320	290.79	22.67	280–400	380.54	20.15
	Ripe	410–580	491.74	37.65	400–600	547.52	12.35
	Overripe	460–610	547.73	47.11	430–700	617.19	89.82
CCN-51	Underripe	280–370	281.86	37.82	300–410	362.78	17.88
	Ripe	390–620	518.82	83.70	400–620	615.50	15.09
	Overripe	460–690	625.85	108.18	410–690	634.55	59.81

NCFA: National Cocoa Fino de Aroma; CCN-51: Colección Castro Naranjal 51. x̅ Weights (g): Average Sample Weight in grams.

**Table 3:** Average weights (g) of cocoa cob components depending on the maturation state at harvest and cocoa variety.

		Dry Season						Rainy Season					
Variety	Maturation	Peel Weight	SD	Almond Weight	SD	Mucilage Weight	SD	Peel Weight	SD	Almond Weight	SD	Mucilage Weight	SD
NCFA	Underripe	138.47	10.80	80.31	6.26	44.31	3.45	173.96	9.21	105.10	5.56	57.99	3.07
	Ripe	211.15	16.19	142.40	10.92	78.57	6.02	191.63	4.32	175.21	3.95	98.55	2.22
	Overripe	224.57	19.32	153.36	13.19	109.55	9.42	253.05	36.83	172.81	25.15	123.44	17.96
CCN-51	Underripe	134.22	18.01	77.85	10.45	42.95	5.76	165.84	8.18	100.20	4.94	55.28	2.73
	Ripe	223.09	35.99	150.46	24.27	83.01	13.39	225.93	5.28	206.56	4.83	116.19	2.72
	Overripe	291.04	61.80	198.76	42.21	141.97	30.15	329.87	59.84	225.27	40.87	160.91	29.19

NCFA: National Cocoa Fino de Aroma; CCN-51: Colección Castro Naranjal 51.

**Table 4:** Physicochemical analysis of the ripe mucilage for elaborating kombucha.

	Variety	
Parameter	CNFA	CCN51
Acidity (%)	1.35	1.44
°Brix	21.58	21.56
pH	3.52	3.34
Humidity (%)	84.53	85.03
Ash (%)	2.45	1.54
Fat (%)	0.36	0.27
Fiber (%)	8.56	8.35
Protein (%)	4.56	4.12
Vitamin C	8.82	8.03
Total Sugars (%)	11.6	13.4
Total Polyphenols (mg)	107.03	67.43

**Table 5:** Results of organoleptic analysis in the fermented beverage. Superscript letters indicate significant differences between the applied treatments (*p* = 0.05).

Sample	Organoleptic Properties					
	Flavor	Color	Texture	Smell	Sweetness	Aftertaste
NCFA100	2.92 ^b^	3.79 ^ab^	3.38 ^ab^	3.25 ^ab^	2.88 ^b^	2.92 ^a^
NCFA80	3.04 ^ab^	3.92 ^a^	3.27 ^ab^	3.27 ^ab^	2.83 ^b^	2.77 ^a^
NCFA60	2.27 ^b^	3.27 ^ab^	2.92 ^b^	3.53 ^ab^	2.23 ^b^	2.83 ^a^
NCFA40	4.84 ^a^	4.24 ^ab^	4.48 ^a^	4.79 ^a^	3.75 ^a^	2.61 ^a^
CCN100	2.92 ^b^	3.79 ^ab^	3.38 ^ab^	3.5 ^ab^	2.88 ^b^	2.92 ^a^
CCN80	3.04 ^ab^	3.92 ^a^	3.27 ^ab^	3.27 ^ab^	2.83 ^b^	2.77 ^a^
CCN60	3.37 ^b^	3.37 ^ab^	3.93 ^b^	3.35 ^ab^	3.33 ^b^	3.83 ^a^
CCN40	3.33 ^b^	3.08 ^b^	3.93 ^b^	3.79 ^b^	3.33 ^b^	3.93 ^a^
Average	2.98	3.57	3.17	3.31	2.88	2.85
C.V. (%)	31.11	24.53	22.97	32.47	34.42	40.54
*p*-value	0.002	0.3307	0.0304	0.054	0.0105	0.9997

**Table 6:** Physicochemical analysis of the finished kombucha product at the time of its production.

Sample	Parameters		
	Acidity (%)	°Brix	pH
NCFA100	0.61 ^b^	12.46 ^ab^	2.20 ^a^
NCFA80	0.49 ^b^	9.31 ^a^	3.25 _b_
NCFA60	0.34 ^b^	7.25 ^ab^	3.63 ^c^
NCFA40	0.20 ^a^	6.12 ^ab^	3.87 ^c^
CCN100	0.72 ^c^	11.96 ^ab^	2.43 ^a^
CCN80	0.51 _b_	8.83 ^a^	3.55 _b_
CCN60	0.33 ^b^	7.27 ^ab^	3.62 ^c^
CCN40	0.17 ^a^	6.78 ^ab^	3.67 ^c^
Average	0.45	8.74	3.47
C.V. (%)	1.11	28.53	16.97
*p*-value	0.002	0.3307	0.0304

Superscript letters indicate significant differences between the applied treatments (*p* = 0.05).

## Data Availability

The original contributions presented in the study are included in the article/Supplementary Material, further inquiries can be directed to the corresponding author.

## Supplementary Materials

The following supporting information can be downloaded at: https://www.mdpi.com/article/10.3390/foods13111636/s1, Anexo S1. Sensory Analysis Format, Anexo S2. Individual weights obtained from cobs and 1 their components. Anexo S3: Figure S1. Results of the organoleptic analysis of parameter color in the fermented drink; Figure S2. Results of the organoleptic analysis of parameter texture in the fermented drink. Figure S3. Results of the organoleptic analysis of parameter Smell in the fermented drink.

## References

[1] Motamayor J.C., Lachenaud P., da Silva e Mota J.W., Loor R., Kuhn D.N., Brown J.S., Schnell R.J. (2008). Geographic and Genetic Population Differentiation of the Amazonian Chocolate Tree *Theobroma cacao* L.. PLoS ONE.

[2] Quingaísa E., Riveros H. (2007). Estudio de Caso: Denominacion de Origen Cacao Arriba.

[3] Almeida A.-A., Valle R.R. (2007). Ecophysiology of the cacao tree. Braz. J. Plant Physiol..

[4] Cruz Chaustre R.A., Cañas Castillo P.C. (2018). La importancia de la exportación del cacao en Colombia y los países en América Latina. Rev. Investig. Gest..

[5] Cedeño C., Otoniel Dilas Jiménez E.J. (2022). Producción y exportación del cacao ecuatoriano y el potencial del cacao fino de aroma. Rev. Investig. Cient. Tecnol. Qantu Yachay.

[6] García Briones A., Pico B., Jaimez R. (2021). La cadena de producción del Cacao en Ecuador: Resiliencia en los diferentes actores de la producción. Rev. Digit. Novasinerg..

[7] García Batista R.M., Quevedo Guerrero J.N., Socorro Castro A.R. (2019). Valoración del estado agronómico de las plantaciones de cacao nacional en el Ecuador. Rev. Metrop. Cienc. Apl..

[8] Anecacao (2023). Tipos de Cacaco—Cacao en el Ecuador.

[9] Nieto Figueroa K.H., Mendoza García N.V., Campos Vega R. (2019). Cocoa By-products. Food Wastes By-Products.

[10] Valdez F. (2021). El cacao fino de aroma, el cacao ancestral emblemático del Ecuador. Am. Lat. IRD Éd..

[11] Girón Guerrero M.F., Márquez Coronel A., Salazar Román E. (2015). Análisis de los Niveles de Desperdicio del Mucílago de Cacao y su Aprovechamiento Como Alternativa de Biocombustible. Bachelor’s Thesis.

[12] Peñaloza Albarracín D.F., Laiton Daza L.J., Caballero Yáñez D.F., BlancoTirado T.d.S., Acevedo Argüello C., Cervantes Díaz M. (2021). Estudio cienciométrico de tendencias en el aprovechamiento de los subproductos del cacao *Theobroma cacao* L.. Rev. Divulg. Cient. Cult. Multidiscip..

[13] Andrade J.A., Rivera-García J., Chire-Fajardo G.C., Ureña-Peralta M.O. (2019). Propiedades físicas y químicas de cultivares de cacao *Theobroma cacao L*. de Ecuador y Perú. SciELO Anal..

[14] Nguyen T., Tien N., Hòa H., Thị N. (2016). A study of wine fermentation from mucilage of Cocoa beans *Theobroma cacao* L.. Dalat Univ. J. Sci..

[15] Cornejo Lucero O.J. (2023). Evaluación del uso de mucilago de cacao *Theobroma cacao* L. como sustrato para la producción de polihidroxialcanoatos. Bachelor’s Thesis.

[16] Anvoh K., Bi A.Z., Gnakri D. (2009). Production and Characterization of Juice from Mucilage of Cocoa Beans and its Transformation into Marmalade. Pak. J. Nutr..

[17] Arana A., Rugel E. (2017). Propuesta de Aprovechamiento del Desecho Mucilago de Cacao en la Hacienda Santa Rita.

[18] Soares T.F., Oliveira M.B.P.P. (2022). Cocoa By-Products: Characterization of Bioactive Compounds and Beneficial Health Effects. Molecules.

[19] Kim-Ngoc V.-T., Cong-Hau N., Bui-Phuc T., Thang N. (2022). Quality Assessment During the Fermentation of Cocoa Beans: Effects of Partial Mucilage Removal. J. Appl. Sci. Environ. Manag..

[20] Puerari C., Magalhães K.T., Schwan R.F. (2012). New cocoa pulp-based kefir beverages: Microbiological, chemical composition and sensory analysis. Food Res. Int..

[21] Barazarte H., Sangronis E., Emaldi U. (2008). La cáscara de cacao *Theobroma cacao* L.: Una posible fuente comercial de pectinas. Arch. Latinoam. Nutr..

[22] Vásquez Z.S., de Carvalho Neto D.P., Pereira G.V.M., Vandenberghe L.P.S., de Oliveira P.Z., Tiburcio P.B., Rogez H.L.G., Góes Neto A., Soccol C.R. (2019). Biotechnological approaches for cocoa waste management: A review. Waste Manag..

[23] Guirlanda C.P., da Silva G.G., Takahashi J.A. (2021). Cocoa honey: Agro-industrial waste or underutilized cocoa by-product?. Futur. Foods.

[24] Arteaga Estrella Y. (2013). Estudio del Desperdicio del Mucilago de Cacao en el Cantón Naranjal Provincia del Guayas.

[25] Yuliana N., Nurainy F., Sari G.W., Sumardi, Widiastuti E.L. (2023). Total microbe, physicochemical property, and antioxidative activity during fermentation of cocoa honey into kombucha functional drink. Appl. Food Res..

[26] Braudeau J. (2012). El Cacao: Técnicas Agricolas y Producciones Tropicales.

[27] Lecumberri E., Mateos R., Izquierdo-Pulido M., Rupérez P., Goya L., Bravo L. (2007). Dietary fibre composition, antioxidant capacity and physico-chemical properties of a fibre-rich product from cocoa (*Theobroma cacao* L.). Food Chem..

[28] Martínez R., Torres P., Meneses M.M., Figueroa J.G., Pérez-Álvarez J.A., Viuda-Martos M. (2012). Chemical, technological and in vitro antioxidant properties of cocoa (*Theobroma cacao* L.) co-products. Food Res. Int..

[29] Montesdeoca S.L., Cepeda J.G., Beltrán J.C., Cruz V.R. (2023). Elaboración de una bebida fermentada utilizando tres variedades de limón (*Citrus limón*). Rev. Cient. Multidiscip. Pentaciencias.

[30] Falcón-Romero P., Aguirre-Vargas E., Asnate-Salazar E. (2021). Elaboración y caracterización de una bebida fermentada elaborada con el fruto de capulí Prunus serotina y miel de abeja. Dominio Cienc..

[31] Haase T.B., Naumann-Gola S., Ortner E., Zorn H., Schweiggert-Weisz U. (2023). Thermal stabilisation of cocoa fruit pulp—Effects on sensory properties, colour and microbiological stability. Curr. Res. Food Sci..

[32] Zou C., Li R.-Y., Chen J.-X., Wang F., Gao Y., Fu Y.-Q., Xu Y.-Q., Yin J.-F. (2021). Zijuan tea- based kombucha: Physicochemical, sensorial, and antioxidant profile. Food Chem..

[33] Rios-Corripio G., Guerrero-Beltrán J. (2020). Physicochemical, Antioxidant and Sensory Characteristics of Black Cherry Prunus Serotina Subsp. Capuli Fermented Juice. Int. J. Fruit Sci..

[34] Santos D., Rezende R., Santos T., Marques E., Ferreira A., Silva A., Romano C., Santos D., Dias J., Tavares Bisneto J. (2020). Fermentation in fine cocoa type Scavina: Change in standard quality as the effect of use of starters yeast in fermentation. Food Chem..

[35] Zhou D.-D., Saimaiti A., Luo M., Huang S.-Y., Xiong R.-G., Shang A., Gan R.-Y., Li H.-B. (2022). Fermentation with Tea Residues Enhances Antioxidant Activities and Polyphenol Contents in Kombucha Beverages. Antioxidants.

[36] Ivanišová E., Meňhartová K., Terentjeva M., Harangozo L., Kántor A., Kačániová M. (2019). The evaluation of chemical, antioxidant, antimicrobial and sensory properties of kombucha tea beverage. J. Food Sci. Technol..

[37] Júnior J.C.d.S., Mafaldo Í.M., Brito I.d.L., Tribuzy de Magalhães Cordeiro A.M. (2022). Kombucha: Formulation, chemical composition, and therapeutic potentialities. Curr. Res. Food Sci..

