# Supplementary material for: Cocoa Mucilage as a Novel Ingredient in Innovative Kombucha Fermentation
Source: Foods. 2024 May 24;13(11):1636. doi: 10.3390/foods13111636 (PMC11171615; doi:10.3390/foods13111636)
Supplement: Supplementary file 1 [file foods-13-01636-s001.zip › foods-2981581-supplementary.pdf]

Anexo S1. Sensory Analysis Format

FORMULARIO DE ANÁLISIS SENSORIAL

Genero: __________

Edad: __________

(de un puntaje del 1 a 5 donde 1 = Bajo y 5 = Alto)

muestra	*Tratamientos*	*Color*	*Sabor*	*Textura*	*Olor*	*Aceptabilidad*
741	CN100					
852	CN80					
963	CN60					
123	CN40					
456	CCC100					
789	CCC80					
357	CCC60					
159	CCC40					

Se ha cambiado la codificación para la evaluación sensorial. La columna de tratamiento no fue visible para los catadores.

Comentarios: ………………

¿Usted compraría este producto si se lanza al mercado?

Si:

No:

¿Porque no?

Gracias

Anexo S2. Individual weights obtained from cobs and their components.

Variety	Season	Maturation	Individual weight of the cocoa pod	Full weight of the cocoa pod	Almond weight (no individual count was made, only weight)	Mucilage weight	Placenta weight
NCFA	Dry	Ripe	460.00	197.80	133.40	73.60	55.20
NCFA	Dry	Ripe	540.00	232.20	156.60	86.40	64.80
NCFA	Dry	Ripe	480.00	206.40	139.20	76.80	57.60
NCFA	Dry	Ripe	453.00	194.79	131.37	72.48	54.36
NCFA	Dry	Ripe	473.00	203.39	137.17	75.68	56.76
NCFA	Dry	Ripe	460.00	197.80	133.40	73.60	55.20
NCFA	Dry	Ripe	540.00	232.20	156.60	86.40	64.80
NCFA	Dry	Ripe	480.00	206.40	139.20	76.80	57.60
NCFA	Dry	Ripe	453.00	194.79	131.37	72.48	54.36
NCFA	Dry	Ripe	476.00	204.68	138.04	76.16	57.12
NCFA	Dry	Ripe	540.00	232.20	156.60	86.40	64.80
NCFA	Dry	Ripe	489.00	210.27	141.81	78.24	58.68
NCFA	Dry	Ripe	453.00	194.79	131.37	72.48	54.36
NCFA	Dry	Ripe	567.00	243.81	164.43	90.72	68.04
NCFA	Dry	Ripe	460.00	197.80	133.40	73.60	55.20
NCFA	Dry	Ripe	540.00	232.20	156.60	86.40	64.80
NCFA	Dry	Ripe	480.00	206.40	139.20	76.80	57.60
NCFA	Dry	Ripe	460.00	197.80	133.40	73.60	55.20
NCFA	Dry	Ripe	540.00	232.20	156.60	86.40	64.80
NCFA	Dry	Ripe	480.00	206.40	139.20	76.80	57.60
NCFA	Dry	Ripe	453.00	194.79	131.37	72.48	54.36
NCFA	Dry	Ripe	473.00	203.39	137.17	75.68	56.76
NCFA	Dry	Ripe	460.00	197.80	133.40	73.60	55.20
NCFA	Dry	Ripe	540.00	232.20	156.60	86.40	64.80
NCFA	Dry	Ripe	480.00	206.40	139.20	76.80	57.60
NCFA	Dry	Ripe	453.00	194.79	131.37	72.48	54.36
NCFA	Dry	Ripe	476.00	204.68	138.04	76.16	57.12
NCFA	Dry	Ripe	540.00	232.20	156.60	86.40	64.80
NCFA	Dry	Ripe	489.00	210.27	141.81	78.24	58.68
NCFA	Dry	Ripe	453.00	194.79	131.37	72.48	54.36
NCFA	Dry	Ripe	567.00	243.81	164.43	90.72	68.04
NCFA	Dry	Ripe	460.00	197.80	133.40	73.60	55.20
NCFA	Dry	Ripe	540.00	232.20	156.60	86.40	64.80
NCFA	Dry	Ripe	480.00	206.40	139.20	76.80	57.60
NCFA	Dry	Ripe	460.00	197.80	133.40	73.60	55.20
NCFA	Dry	Ripe	540.00	232.20	156.60	86.40	64.80
NCFA	Dry	Ripe	480.00	206.40	139.20	76.80	57.60
NCFA	Dry	Ripe	453.00	194.79	131.37	72.48	54.36
NCFA	Dry	Ripe	473.00	203.39	137.17	75.68	56.76
NCFA	Dry	Ripe	460.00	197.80	133.40	73.60	55.20
NCFA	Dry	Ripe	540.00	232.20	156.60	86.40	64.80
NCFA	Dry	Ripe	480.00	206.40	139.20	76.80	57.60
NCFA	Dry	Ripe	453.00	194.79	131.37	72.48	54.36
NCFA	Dry	Ripe	476.00	204.68	138.04	76.16	57.12
NCFA	Dry	Ripe	540.00	232.20	156.60	86.40	64.80
NCFA	Dry	Ripe	489.00	210.27	141.81	78.24	58.68
NCFA	Dry	Ripe	453.00	194.79	131.37	72.48	54.36
NCFA	Dry	Ripe	567.00	243.81	164.43	90.72	68.04
NCFA	Dry	Ripe	460.00	197.80	133.40	73.60	55.20
NCFA	Dry	Ripe	540.00	232.20	156.60	86.40	64.80
NCFA	Rainy	Ripe	540.00	189.00	172.80	97.20	81.00

NCFA	Rainy	Ripe	550.00	192.50	176.00	99.00	82.50
NCFA	Rainy	Ripe	530.00	185.50	169.60	95.40	79.50
NCFA	Rainy	Ripe	553.00	193.55	176.96	99.54	82.95
NCFA	Rainy	Ripe	573.00	200.55	183.36	103.14	85.95
NCFA	Rainy	Ripe	540.00	189.00	172.80	97.20	81.00
NCFA	Rainy	Ripe	550.00	192.50	176.00	99.00	82.50
NCFA	Rainy	Ripe	530.00	185.50	169.60	95.40	79.50
NCFA	Rainy	Ripe	553.00	193.55	176.96	99.54	82.95
NCFA	Rainy	Ripe	574.00	200.90	183.68	103.32	86.10
NCFA	Rainy	Ripe	550.00	192.50	176.00	99.00	82.50
NCFA	Rainy	Ripe	539.00	188.65	172.48	97.02	80.85
NCFA	Rainy	Ripe	553.00	193.55	176.96	99.54	82.95
NCFA	Rainy	Ripe	547.00	191.45	175.04	98.46	82.05
NCFA	Rainy	Ripe	540.00	189.00	172.80	97.20	81.00
NCFA	Rainy	Ripe	550.00	192.50	176.00	99.00	82.50
NCFA	Rainy	Ripe	530.00	185.50	169.60	95.40	79.50
NCFA	Rainy	Ripe	540.00	189.00	172.80	97.20	81.00
NCFA	Rainy	Ripe	550.00	192.50	176.00	99.00	82.50
NCFA	Rainy	Ripe	530.00	185.50	169.60	95.40	79.50
NCFA	Rainy	Ripe	553.00	193.55	176.96	99.54	82.95
NCFA	Rainy	Ripe	573.00	200.55	183.36	103.14	85.95
NCFA	Rainy	Ripe	540.00	189.00	172.80	97.20	81.00
NCFA	Rainy	Ripe	550.00	192.50	176.00	99.00	82.50
NCFA	Rainy	Ripe	530.00	185.50	169.60	95.40	79.50
NCFA	Rainy	Ripe	553.00	193.55	176.96	99.54	82.95
NCFA	Rainy	Ripe	574.00	200.90	183.68	103.32	86.10
NCFA	Rainy	Ripe	550.00	192.50	176.00	99.00	82.50
NCFA	Rainy	Ripe	539.00	188.65	172.48	97.02	80.85
NCFA	Rainy	Ripe	553.00	193.55	176.96	99.54	82.95
NCFA	Rainy	Ripe	547.00	191.45	175.04	98.46	82.05
NCFA	Rainy	Ripe	540.00	189.00	172.80	97.20	81.00
NCFA	Rainy	Ripe	550.00	192.50	176.00	99.00	82.50
NCFA	Rainy	Ripe	530.00	185.50	169.60	95.40	79.50
NCFA	Rainy	Ripe	540.00	189.00	172.80	97.20	81.00
NCFA	Rainy	Ripe	550.00	192.50	176.00	99.00	82.50
NCFA	Rainy	Ripe	530.00	185.50	169.60	95.40	79.50
NCFA	Rainy	Ripe	553.00	193.55	176.96	99.54	82.95
NCFA	Rainy	Ripe	573.00	200.55	183.36	103.14	85.95
NCFA	Rainy	Ripe	540.00	189.00	172.80	97.20	81.00
NCFA	Rainy	Ripe	550.00	192.50	176.00	99.00	82.50
NCFA	Rainy	Ripe	530.00	185.50	169.60	95.40	79.50
NCFA	Rainy	Ripe	553.00	193.55	176.96	99.54	82.95
NCFA	Rainy	Ripe	574.00	200.90	183.68	103.32	86.10
NCFA	Rainy	Ripe	550.00	192.50	176.00	99.00	82.50
NCFA	Rainy	Ripe	539.00	188.65	172.48	97.02	80.85
NCFA	Rainy	Ripe	553.00	193.55	176.96	99.54	82.95
NCFA	Rainy	Ripe	547.00	191.45	175.04	98.46	82.05
NCFA	Rainy	Ripe	540.00	189.00	172.80	97.20	81.00
NCFA	Rainy	Ripe	550.00	192.50	176.00	99.00	82.50
CCN-51	Dry	Ripe	460.00	197.80	133.40	73.60	55.20
CCN-51	Dry	Ripe	640.00	275.20	185.60	102.40	76.80
CCN-51	Dry	Ripe	480.00	206.40	139.20	76.80	57.60
CCN-51	Dry	Ripe	462.00	198.66	133.98	73.92	55.44
CCN-51	Dry	Ripe	442.00	190.06	128.18	70.72	53.04
CCN-51	Dry	Ripe	460.00	197.80	133.40	73.60	55.20
CCN-51	Dry	Ripe	640.00	275.20	185.60	102.40	76.80
CCN-51	Dry	Ripe	480.00	206.40	139.20	76.80	57.60
CCN-51	Dry	Ripe	462.00	198.66	133.98	73.92	55.44

CCN-51	Dry	Ripe	446.00	191.78	129.34	71.36	53.52
CCN-51	Dry	Ripe	640.00	275.20	185.60	102.40	76.80
CCN-51	Dry	Ripe	489.00	210.27	141.81	78.24	58.68
CCN-51	Dry	Ripe	462.00	198.66	133.98	73.92	55.44
CCN-51	Dry	Ripe	664.00	285.52	192.56	106.24	79.68
CCN-51	Dry	Ripe	460.00	197.80	133.40	73.60	55.20
CCN-51	Dry	Ripe	640.00	275.20	185.60	102.40	76.80
CCN-51	Dry	Ripe	480.00	206.40	139.20	76.80	57.60
CCN-51	Dry	Ripe	460.00	197.80	133.40	73.60	55.20
CCN-51	Dry	Ripe	640.00	275.20	185.60	102.40	76.80
CCN-51	Dry	Ripe	480.00	206.40	139.20	76.80	57.60
CCN-51	Dry	Ripe	462.00	198.66	133.98	73.92	55.44
CCN-51	Dry	Ripe	442.00	190.06	128.18	70.72	53.04
CCN-51	Dry	Ripe	460.00	197.80	133.40	73.60	55.20
CCN-51	Dry	Ripe	640.00	275.20	185.60	102.40	76.80
CCN-51	Dry	Ripe	480.00	206.40	139.20	76.80	57.60
CCN-51	Dry	Ripe	462.00	198.66	133.98	73.92	55.44
CCN-51	Dry	Ripe	446.00	191.78	129.34	71.36	53.52
CCN-51	Dry	Ripe	640.00	275.20	185.60	102.40	76.80
CCN-51	Dry	Ripe	489.00	210.27	141.81	78.24	58.68
CCN-51	Dry	Ripe	462.00	198.66	133.98	73.92	55.44
CCN-51	Dry	Ripe	664.00	285.52	192.56	106.24	79.68
CCN-51	Dry	Ripe	460.00	197.80	133.40	73.60	55.20
CCN-51	Dry	Ripe	640.00	275.20	185.60	102.40	76.80
CCN-51	Dry	Ripe	480.00	206.40	139.20	76.80	57.60
CCN-51	Dry	Ripe	460.00	197.80	133.40	73.60	55.20
CCN-51	Dry	Ripe	640.00	275.20	185.60	102.40	76.80
CCN-51	Dry	Ripe	480.00	206.40	139.20	76.80	57.60
CCN-51	Dry	Ripe	462.00	198.66	133.98	73.92	55.44
CCN-51	Dry	Ripe	442.00	190.06	128.18	70.72	53.04
CCN-51	Dry	Ripe	460.00	197.80	133.40	73.60	55.20
CCN-51	Dry	Ripe	640.00	275.20	185.60	102.40	76.80
CCN-51	Dry	Ripe	480.00	206.40	139.20	76.80	57.60
CCN-51	Dry	Ripe	462.00	198.66	133.98	73.92	55.44
CCN-51	Dry	Ripe	446.00	191.78	129.34	71.36	53.52
CCN-51	Dry	Ripe	640.00	275.20	185.60	102.40	76.80
CCN-51	Dry	Ripe	489.00	210.27	141.81	78.24	58.68
CCN-51	Dry	Ripe	462.00	198.66	133.98	73.92	55.44
CCN-51	Dry	Ripe	664.00	285.52	192.56	106.24	79.68
CCN-51	Dry	Ripe	460.00	197.80	133.40	73.60	55.20
CCN-51	Dry	Ripe	640.00	275.20	185.60	102.40	76.80
CCN-51	Rainy	Ripe	640.00	224.00	204.80	115.20	96.00
CCN-51	Rainy	Ripe	660.00	231.00	211.20	118.80	99.00
CCN-51	Rainy	Ripe	620.00	217.00	198.40	111.60	93.00
CCN-51	Rainy	Ripe	662.00	231.70	211.84	119.16	99.30

CCN-51	Rainy	Ripe	642.00	224.70	205.44	115.56	96.30
CCN-51	Rainy	Ripe	640.00	224.00	204.80	115.20	96.00
CCN-51	Rainy	Ripe	660.00	231.00	211.20	118.80	99.00
CCN-51	Rainy	Ripe	620.00	217.00	198.40	111.60	93.00
CCN-51	Rainy	Ripe	662.00	231.70	211.84	119.16	99.30
CCN-51	Rainy	Ripe	644.00	225.40	206.08	115.92	96.60
CCN-51	Rainy	Ripe	660.00	231.00	211.20	118.80	99.00
CCN-51	Rainy	Ripe	629.00	220.15	201.28	113.22	94.35
CCN-51	Rainy	Ripe	662.00	231.70	211.84	119.16	99.30
CCN-51	Rainy	Ripe	644.00	225.40	206.08	115.92	96.60
CCN-51	Rainy	Ripe	640.00	224.00	204.80	115.20	96.00
CCN-51	Rainy	Ripe	660.00	231.00	211.20	118.80	99.00
CCN-51	Rainy	Ripe	620.00	217.00	198.40	111.60	93.00
CCN-51	Rainy	Ripe	640.00	224.00	204.80	115.20	96.00
CCN-51	Rainy	Ripe	660.00	231.00	211.20	118.80	99.00
CCN-51	Rainy	Ripe	620.00	217.00	198.40	111.60	93.00
CCN-51	Rainy	Ripe	662.00	231.70	211.84	119.16	99.30
CCN-51	Rainy	Ripe	642.00	224.70	205.44	115.56	96.30
CCN-51	Rainy	Ripe	640.00	224.00	204.80	115.20	96.00
CCN-51	Rainy	Ripe	660.00	231.00	211.20	118.80	99.00
CCN-51	Rainy	Ripe	620.00	217.00	198.40	111.60	93.00
CCN-51	Rainy	Ripe	662.00	231.70	211.84	119.16	99.30
CCN-51	Rainy	Ripe	644.00	225.40	206.08	115.92	96.60
CCN-51	Rainy	Ripe	660.00	231.00	211.20	118.80	99.00
CCN-51	Rainy	Ripe	629.00	220.15	201.28	113.22	94.35
CCN-51	Rainy	Ripe	662.00	231.70	211.84	119.16	99.30
CCN-51	Rainy	Ripe	644.00	225.40	206.08	115.92	96.60
CCN-51	Rainy	Ripe	640.00	224.00	204.80	115.20	96.00
CCN-51	Rainy	Ripe	660.00	231.00	211.20	118.80	99.00
CCN-51	Rainy	Ripe	620.00	217.00	198.40	111.60	93.00
CCN-51	Rainy	Ripe	640.00	224.00	204.80	115.20	96.00
CCN-51	Rainy	Ripe	660.00	231.00	211.20	118.80	99.00
CCN-51	Rainy	Ripe	620.00	217.00	198.40	111.60	93.00
CCN-51	Rainy	Ripe	662.00	231.70	211.84	119.16	99.30
CCN-51	Rainy	Ripe	642.00	224.70	205.44	115.56	96.30
CCN-51	Rainy	Ripe	640.00	224.00	204.80	115.20	96.00
CCN-51	Rainy	Ripe	660.00	231.00	211.20	118.80	99.00
CCN-51	Rainy	Ripe	620.00	217.00	198.40	111.60	93.00
CCN-51	Rainy	Ripe	662.00	231.70	211.84	119.16	99.30
CCN-51	Rainy	Ripe	644.00	225.40	206.08	115.92	96.60
CCN-51	Rainy	Ripe	660.00	231.00	211.20	118.80	99.00
CCN-51	Rainy	Ripe	629.00	220.15	201.28	113.22	94.35
CCN-51	Rainy	Ripe	662.00	231.70	211.84	119.16	99.30
CCN-51	Rainy	Ripe	644.00	225.40	206.08	115.92	96.60
CCN-51	Rainy	Ripe	640.00	224.00	204.80	115.20	96.00

CCN-51	Rainy	Ripe	660.00	231.00	211.20	118.80	99.00
NCFA	Dry	Over-Ripe	567.00	232.47	158.76	113.40	62.37
NCFA	Dry	Over-Ripe	460.00	188.60	128.80	92.00	50.60
NCFA	Dry	Over-Ripe	540.00	221.40	151.20	108.00	59.40
NCFA	Dry	Over-Ripe	540.00	221.40	151.20	108.00	59.40
NCFA	Dry	Over-Ripe	567.00	232.47	158.76	113.40	62.37
NCFA	Dry	Over-Ripe	567.00	232.47	158.76	113.40	62.37
NCFA	Dry	Over-Ripe	460.00	188.60	128.80	92.00	50.60
NCFA	Dry	Over-Ripe	540.00	221.40	151.20	108.00	59.40
NCFA	Dry	Over-Ripe	525.79	215.57	147.22	105.16	57.84
NCFA	Dry	Over-Ripe	587.00	240.67	164.36	117.40	64.57
NCFA	Dry	Over-Ripe	544.00	223.04	152.32	108.80	59.84
NCFA	Dry	Over-Ripe	565.00	231.65	158.20	113.00	62.15
NCFA	Dry	Over-Ripe	543.00	222.63	152.04	108.60	59.73
NCFA	Dry	Over-Ripe	567.00	232.47	158.76	113.40	62.37
NCFA	Dry	Over-Ripe	520.00	213.20	145.60	104.00	57.20
NCFA	Dry	Over-Ripe	587.00	240.67	164.36	117.40	64.57
NCFA	Dry	Over-Ripe	540.00	221.40	151.20	108.00	59.40
NCFA	Dry	Over-Ripe	567.00	232.47	158.76	113.40	62.37
NCFA	Dry	Over-Ripe	567.00	232.47	158.76	113.40	62.37
NCFA	Dry	Over-Ripe	460.00	188.60	128.80	92.00	50.60
NCFA	Dry	Over-Ripe	540.00	221.40	151.20	108.00	59.40
NCFA	Dry	Over-Ripe	521.93	213.99	146.14	104.39	57.41
NCFA	Dry	Over-Ripe	520.96	213.60	145.87	104.19	57.31
NCFA	Dry	Over-Ripe	588.00	241.08	164.64	117.60	64.68
NCFA	Dry	Over-Ripe	519.04	212.80	145.33	103.81	57.09
NCFA	Dry	Over-Ripe	520.96	213.60	145.87	104.19	57.31
NCFA	Dry	Over-Ripe	520.00	213.20	145.60	104.00	57.20
NCFA	Dry	Over-Ripe	519.04	212.80	145.33	103.81	57.09
NCFA	Dry	Over-Ripe	540.00	221.40	151.20	108.00	59.40
NCFA	Dry	Over-Ripe	567.00	232.47	158.76	113.40	62.37
NCFA	Dry	Over-Ripe	567.00	232.47	158.76	113.40	62.37
NCFA	Dry	Over-Ripe	460.00	188.60	128.80	92.00	50.60
NCFA	Dry	Over-Ripe	540.00	221.40	151.20	108.00	59.40
NCFA	Dry	Over-Ripe	521.93	213.99	146.14	104.39	57.41
NCFA	Dry	Over-Ripe	567.00	232.47	158.76	113.40	62.37
NCFA	Dry	Over-Ripe	520.00	213.20	145.60	104.00	57.20
NCFA	Dry	Over-Ripe	599.00	245.59	167.72	119.80	65.89
NCFA	Dry	Over-Ripe	520.00	213.20	145.60	104.00	57.20
NCFA	Dry	Over-Ripe	615.00	252.15	172.20	123.00	67.65
NCFA	Dry	Over-Ripe	540.00	221.40	151.20	108.00	59.40
NCFA	Dry	Over-Ripe	567.00	232.47	158.76	113.40	62.37
NCFA	Dry	Over-Ripe	567.00	232.47	158.76	113.40	62.37
NCFA	Dry	Over-Ripe	460.00	188.60	128.80	92.00	50.60
NCFA	Dry	Over-Ripe	540.00	221.40	151.20	108.00	59.40

NCFA	Dry	Over-Ripe	521.93	213.99	146.14	104.39	57.41
NCFA	Dry	Over-Ripe	688.00	282.08	192.64	137.60	75.68
NCFA	Dry	Over-Ripe	675.00	276.75	189.00	135.00	74.25
NCFA	Dry	Over-Ripe	540.00	221.40	151.20	108.00	59.40
NCFA	Dry	Over-Ripe	567.00	232.47	158.76	113.40	62.37
NCFA	Dry	Over-Ripe	640.00	262.40	179.20	128.00	70.40
NCFA	Rainy	Over-Ripe	667.00	273.47	186.76	133.40	73.37
NCFA	Rainy	Over-Ripe	360.00	147.60	100.80	72.00	39.60
NCFA	Rainy	Over-Ripe	630.00	258.30	176.40	126.00	69.30
NCFA	Rainy	Over-Ripe	630.00	258.30	176.40	126.00	69.30
NCFA	Rainy	Over-Ripe	667.00	273.47	186.76	133.40	73.37
NCFA	Rainy	Over-Ripe	667.00	273.47	186.76	133.40	73.37
NCFA	Rainy	Over-Ripe	360.00	147.60	100.80	72.00	39.60
NCFA	Rainy	Over-Ripe	630.00	258.30	176.40	126.00	69.30
NCFA	Rainy	Over-Ripe	626.79	256.98	175.50	125.36	68.95
NCFA	Rainy	Over-Ripe	687.00	281.67	192.36	137.40	75.57
NCFA	Rainy	Over-Ripe	633.00	259.53	177.24	126.60	69.63
NCFA	Rainy	Over-Ripe	666.00	273.06	186.48	133.20	73.26
NCFA	Rainy	Over-Ripe	633.00	259.53	177.24	126.60	69.63
NCFA	Rainy	Over-Ripe	667.00	273.47	186.76	133.40	73.37
NCFA	Rainy	Over-Ripe	620.00	254.20	173.60	124.00	68.20
NCFA	Rainy	Over-Ripe	687.00	281.67	192.36	137.40	75.57
NCFA	Rainy	Over-Ripe	630.00	258.30	176.40	126.00	69.30
NCFA	Rainy	Over-Ripe	667.00	273.47	186.76	133.40	73.37
NCFA	Rainy	Over-Ripe	667.00	273.47	186.76	133.40	73.37
NCFA	Rainy	Over-Ripe	360.00	147.60	100.80	72.00	39.60
NCFA	Rainy	Over-Ripe	630.00	258.30	176.40	126.00	69.30
NCFA	Rainy	Over-Ripe	621.93	254.99	174.14	124.39	68.41
NCFA	Rainy	Over-Ripe	620.96	254.59	173.87	124.19	68.31
NCFA	Rainy	Over-Ripe	688.00	282.08	192.64	137.60	75.68
NCFA	Rainy	Over-Ripe	619.04	253.81	173.33	123.81	68.09
NCFA	Rainy	Over-Ripe	620.96	254.59	173.87	124.19	68.31
NCFA	Rainy	Over-Ripe	620.00	254.20	173.60	124.00	68.20
NCFA	Rainy	Over-Ripe	619.04	253.81	173.33	123.81	68.09
NCFA	Rainy	Over-Ripe	630.00	258.30	176.40	126.00	69.30
NCFA	Rainy	Over-Ripe	667.00	273.47	186.76	133.40	73.37
NCFA	Rainy	Over-Ripe	667.00	273.47	186.76	133.40	73.37
NCFA	Rainy	Over-Ripe	360.00	147.60	100.80	72.00	39.60
NCFA	Rainy	Over-Ripe	630.00	258.30	176.40	126.00	69.30
NCFA	Rainy	Over-Ripe	621.93	254.99	174.14	124.39	68.41
NCFA	Rainy	Over-Ripe	667.00	273.47	186.76	133.40	73.37
NCFA	Rainy	Over-Ripe	620.00	254.20	173.60	124.00	68.20
NCFA	Rainy	Over-Ripe	699.00	286.59	195.72	139.80	76.89
NCFA	Rainy	Over-Ripe	620.00	254.20	173.60	124.00	68.20
NCFA	Rainy	Over-Ripe	616.00	252.56	172.48	123.20	67.76

NCFA	Rainy	Over-Ripe	630.00	258.30	176.40	126.00	69.30
NCFA	Rainy	Over-Ripe	667.00	273.47	186.76	133.40	73.37
NCFA	Rainy	Over-Ripe	667.00	273.47	186.76	133.40	73.37
NCFA	Rainy	Over-Ripe	360.00	147.60	100.80	72.00	39.60
NCFA	Rainy	Over-Ripe	630.00	258.30	176.40	126.00	69.30
NCFA	Rainy	Over-Ripe	621.93	254.99	174.14	124.39	68.41
NCFA	Rainy	Over-Ripe	688.00	282.08	192.64	137.60	75.68
NCFA	Rainy	Over-Ripe	676.00	277.16	189.28	135.20	74.36
NCFA	Rainy	Over-Ripe	630.00	258.30	176.40	126.00	69.30
NCFA	Rainy	Over-Ripe	667.00	273.47	186.76	133.40	73.37
NCFA	Rainy	Over-Ripe	630.00	258.30	176.40	126.00	69.30
CCN-51	Dry	Over-Ripe	787.00	322.67	220.36	157.40	86.57
CCN-51	Dry	Over-Ripe	280.00	114.80	78.40	56.00	30.80
CCN-51	Dry	Over-Ripe	720.00	295.20	201.60	144.00	79.20
CCN-51	Dry	Over-Ripe	720.00	295.20	201.60	144.00	79.20
CCN-51	Dry	Over-Ripe	787.00	322.67	220.36	157.40	86.57
CCN-51	Dry	Over-Ripe	787.00	322.67	220.36	157.40	86.57
CCN-51	Dry	Over-Ripe	280.00	114.80	78.40	56.00	30.80
CCN-51	Dry	Over-Ripe	720.00	295.20	201.60	144.00	79.20
CCN-51	Dry	Over-Ripe	727.79	298.39	203.78	145.56	80.06
CCN-51	Dry	Over-Ripe	787.00	322.67	220.36	157.40	86.57
CCN-51	Dry	Over-Ripe	722.00	296.02	202.16	144.40	79.42
CCN-51	Dry	Over-Ripe	787.00	322.67	220.36	157.40	86.57
CCN-51	Dry	Over-Ripe	723.00	296.43	202.44	144.60	79.53
CCN-51	Dry	Over-Ripe	787.00	322.67	220.36	157.40	86.57
CCN-51	Dry	Over-Ripe	720.00	295.20	201.60	144.00	79.20
CCN-51	Dry	Over-Ripe	787.00	322.67	220.36	157.40	86.57
CCN-51	Dry	Over-Ripe	720.00	295.20	201.60	144.00	79.20
CCN-51	Dry	Over-Ripe	787.00	322.67	220.36	157.40	86.57
CCN-51	Dry	Over-Ripe	787.00	322.67	220.36	157.40	86.57
CCN-51	Dry	Over-Ripe	280.00	114.80	78.40	56.00	30.80
CCN-51	Dry	Over-Ripe	720.00	295.20	201.60	144.00	79.20
CCN-51	Dry	Over-Ripe	721.93	295.99	202.14	144.39	79.41
CCN-51	Dry	Over-Ripe	720.98	295.60	201.88	144.20	79.31
CCN-51	Dry	Over-Ripe	788.00	323.08	220.64	157.60	86.68
CCN-51	Dry	Over-Ripe	719.04	294.81	201.33	143.81	79.09
CCN-51	Dry	Over-Ripe	720.98	295.60	201.88	144.20	79.31
CCN-51	Dry	Over-Ripe	720.00	295.20	201.60	144.00	79.20
CCN-51	Dry	Over-Ripe	719.04	294.81	201.33	143.81	79.09
CCN-51	Dry	Over-Ripe	720.00	295.20	201.60	144.00	79.20
CCN-51	Dry	Over-Ripe	787.00	322.67	220.36	157.40	86.57
CCN-51	Dry	Over-Ripe	787.00	322.67	220.36	157.40	86.57
CCN-51	Dry	Over-Ripe	280.00	114.80	78.40	56.00	30.80
CCN-51	Dry	Over-Ripe	720.00	295.20	201.60	144.00	79.20
CCN-51	Dry	Over-Ripe	721.93	295.99	202.14	144.39	79.41

CCN-51	Dry	Over-Ripe	787.00	322.67	220.36	157.40	86.57
CCN-51	Dry	Over-Ripe	720.00	295.20	201.60	144.00	79.20
CCN-51	Dry	Over-Ripe	799.00	327.59	223.72	159.80	87.89
CCN-51	Dry	Over-Ripe	720.00	295.20	201.60	144.00	79.20
CCN-51	Dry	Over-Ripe	817.00	334.97	228.76	163.40	89.87
CCN-51	Dry	Over-Ripe	720.00	295.20	201.60	144.00	79.20
CCN-51	Dry	Over-Ripe	787.00	322.67	220.36	157.40	86.57
CCN-51	Dry	Over-Ripe	787.00	322.67	220.36	157.40	86.57
CCN-51	Dry	Over-Ripe	280.00	114.80	78.40	56.00	30.80
CCN-51	Dry	Over-Ripe	720.00	295.20	201.60	144.00	79.20
CCN-51	Dry	Over-Ripe	721.93	295.99	202.14	144.39	79.41
CCN-51	Dry	Over-Ripe	888.00	364.08	248.64	177.60	97.68
CCN-51	Dry	Over-Ripe	877.00	359.57	245.56	175.40	96.47
CCN-51	Dry	Over-Ripe	720.00	295.20	201.60	144.00	79.20
CCN-51	Dry	Over-Ripe	787.00	322.67	220.36	157.40	86.57
CCN-51	Dry	Over-Ripe	820.00	336.20	229.60	164.00	90.20
CCN-51	Rainy	Over-Ripe	887.00	363.67	248.36	177.40	97.57
CCN-51	Rainy	Over-Ripe	380.00	155.80	106.40	76.00	41.80
CCN-51	Rainy	Over-Ripe	830.00	340.30	232.40	166.00	91.30
CCN-51	Rainy	Over-Ripe	830.00	340.30	232.40	166.00	91.30
CCN-51	Rainy	Over-Ripe	887.00	363.67	248.36	177.40	97.57
CCN-51	Rainy	Over-Ripe	887.00	363.67	248.36	177.40	97.57
CCN-51	Rainy	Over-Ripe	380.00	155.80	106.40	76.00	41.80
CCN-51	Rainy	Over-Ripe	830.00	340.30	232.40	166.00	91.30
CCN-51	Rainy	Over-Ripe	828.79	339.80	232.06	165.76	91.17
CCN-51	Rainy	Over-Ripe	887.00	363.67	248.36	177.40	97.57
CCN-51	Rainy	Over-Ripe	833.00	341.53	233.24	166.60	91.63
CCN-51	Rainy	Over-Ripe	888.00	364.08	248.64	177.60	97.68
CCN-51	Rainy	Over-Ripe	833.00	341.53	233.24	166.60	91.63
CCN-51	Rainy	Over-Ripe	887.00	363.67	248.36	177.40	97.57
CCN-51	Rainy	Over-Ripe	820.00	336.20	229.60	164.00	90.20
CCN-51	Rainy	Over-Ripe	887.00	363.67	248.36	177.40	97.57
CCN-51	Rainy	Over-Ripe	830.00	340.30	232.40	166.00	91.30
CCN-51	Rainy	Over-Ripe	887.00	363.67	248.36	177.40	97.57
CCN-51	Rainy	Over-Ripe	887.00	363.67	248.36	177.40	97.57
CCN-51	Rainy	Over-Ripe	380.00	155.80	106.40	76.00	41.80
CCN-51	Rainy	Over-Ripe	830.00	340.30	232.40	166.00	91.30
CCN-51	Rainy	Over-Ripe	821.93	336.99	230.14	164.39	90.41
CCN-51	Rainy	Over-Ripe	820.98	336.60	229.88	164.20	90.31
CCN-51	Rainy	Over-Ripe	888.00	364.08	248.64	177.60	97.68
CCN-51	Rainy	Over-Ripe	819.04	335.81	229.33	163.81	90.09
CCN-51	Rainy	Over-Ripe	820.98	336.60	229.88	164.20	90.31
CCN-51	Rainy	Over-Ripe	820.00	336.20	229.60	164.00	90.20
CCN-51	Rainy	Over-Ripe	819.04	335.81	229.33	163.81	90.09
CCN-51	Rainy	Over-Ripe	830.00	340.30	232.40	166.00	91.30

CCN-51	Rainy	Over-Ripe	887.00	363.67	248.36	177.40	97.57
CCN-51	Rainy	Over-Ripe	887.00	363.67	248.36	177.40	97.57
CCN-51	Rainy	Over-Ripe	380.00	155.80	106.40	76.00	41.80
CCN-51	Rainy	Over-Ripe	830.00	340.30	232.40	166.00	91.30
CCN-51	Rainy	Over-Ripe	821.93	336.99	230.14	164.39	90.41
CCN-51	Rainy	Over-Ripe	887.00	363.67	248.36	177.40	97.57
CCN-51	Rainy	Over-Ripe	820.00	336.20	229.60	164.00	90.20
CCN-51	Rainy	Over-Ripe	899.00	368.59	251.72	179.80	98.89
CCN-51	Rainy	Over-Ripe	820.00	336.20	229.60	164.00	90.20
CCN-51	Rainy	Over-Ripe	818.00	335.38	229.04	163.60	89.98
CCN-51	Rainy	Over-Ripe	830.00	340.30	232.40	166.00	91.30
CCN-51	Rainy	Over-Ripe	887.00	363.67	248.36	177.40	97.57
CCN-51	Rainy	Over-Ripe	887.00	363.67	248.36	177.40	97.57
CCN-51	Rainy	Over-Ripe	380.00	155.80	106.40	76.00	41.80
CCN-51	Rainy	Over-Ripe	830.00	340.30	232.40	166.00	91.30
CCN-51	Rainy	Over-Ripe	821.93	336.99	230.14	164.39	90.41
CCN-51	Rainy	Over-Ripe	888.00	364.08	248.64	177.60	97.68
CCN-51	Rainy	Over-Ripe	878.00	359.98	245.84	175.60	96.58
CCN-51	Rainy	Over-Ripe	830.00	340.30	232.40	166.00	91.30
CCN-51	Rainy	Over-Ripe	887.00	363.67	248.36	177.40	97.57
CCN-51	Rainy	Over-Ripe	830.00	340.30	232.40	166.00	91.30

NCFA**:** National Cocoa Fino de Aroma; CCN-51: Colección Castro Naranjal 51

Anexo S3

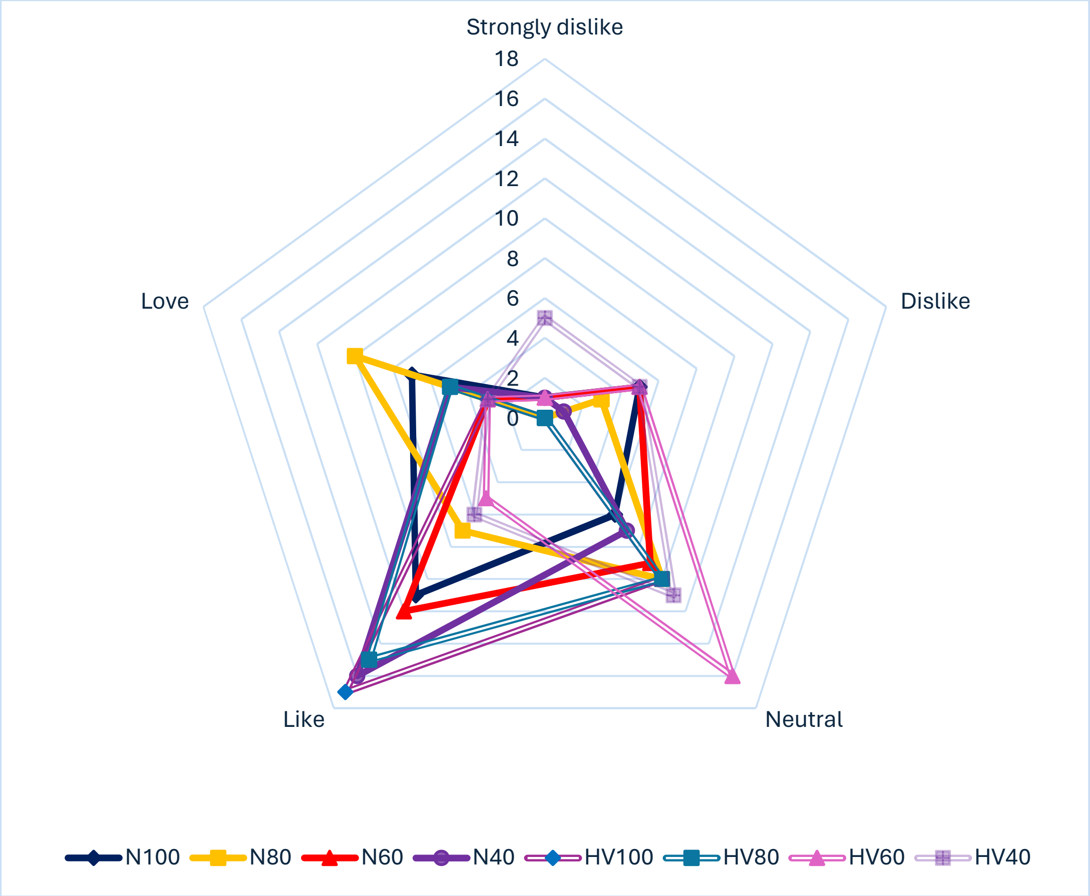

Figure S1. Results of the organoleptic analysis of parameter color in the fermented drink.

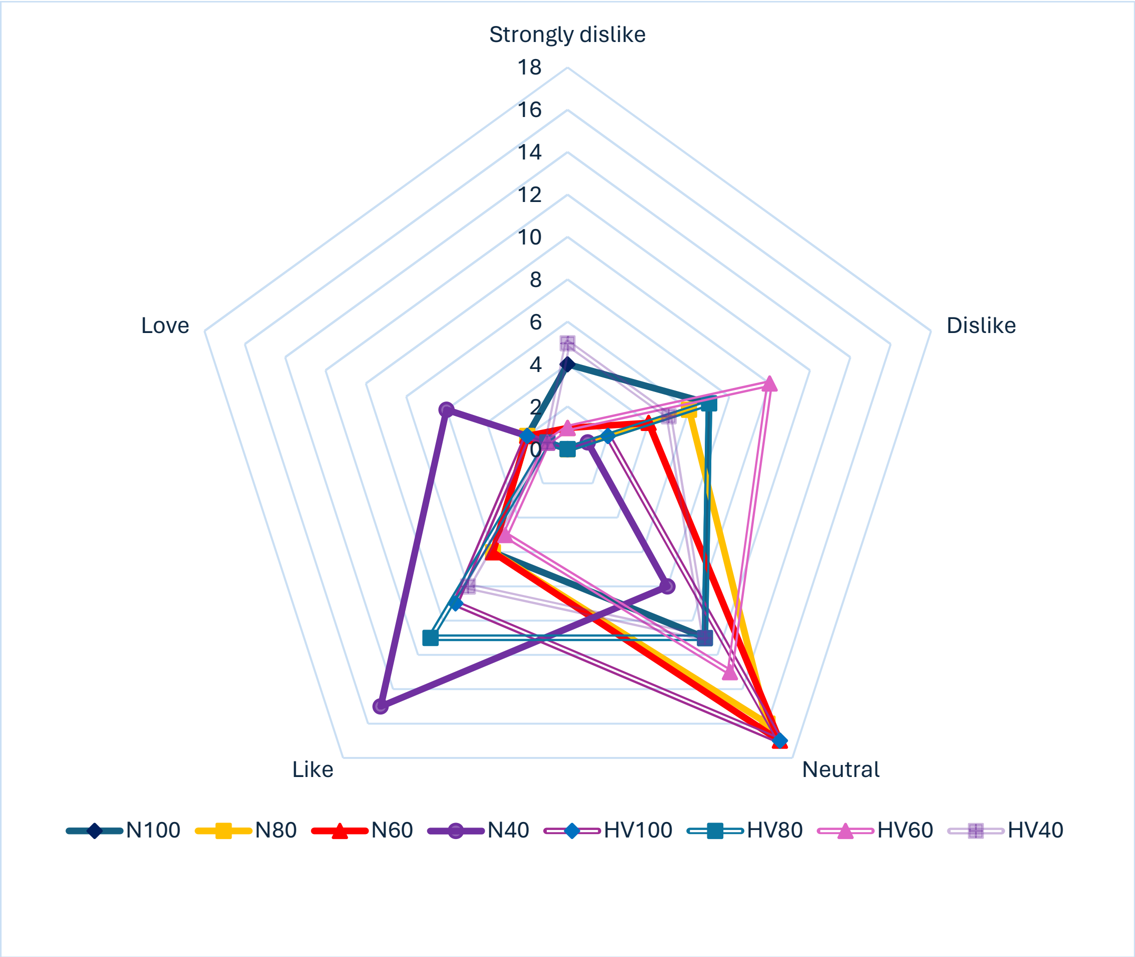

Figure S2.Results of the organoleptic analysis of parameter texture in the fermented drink.

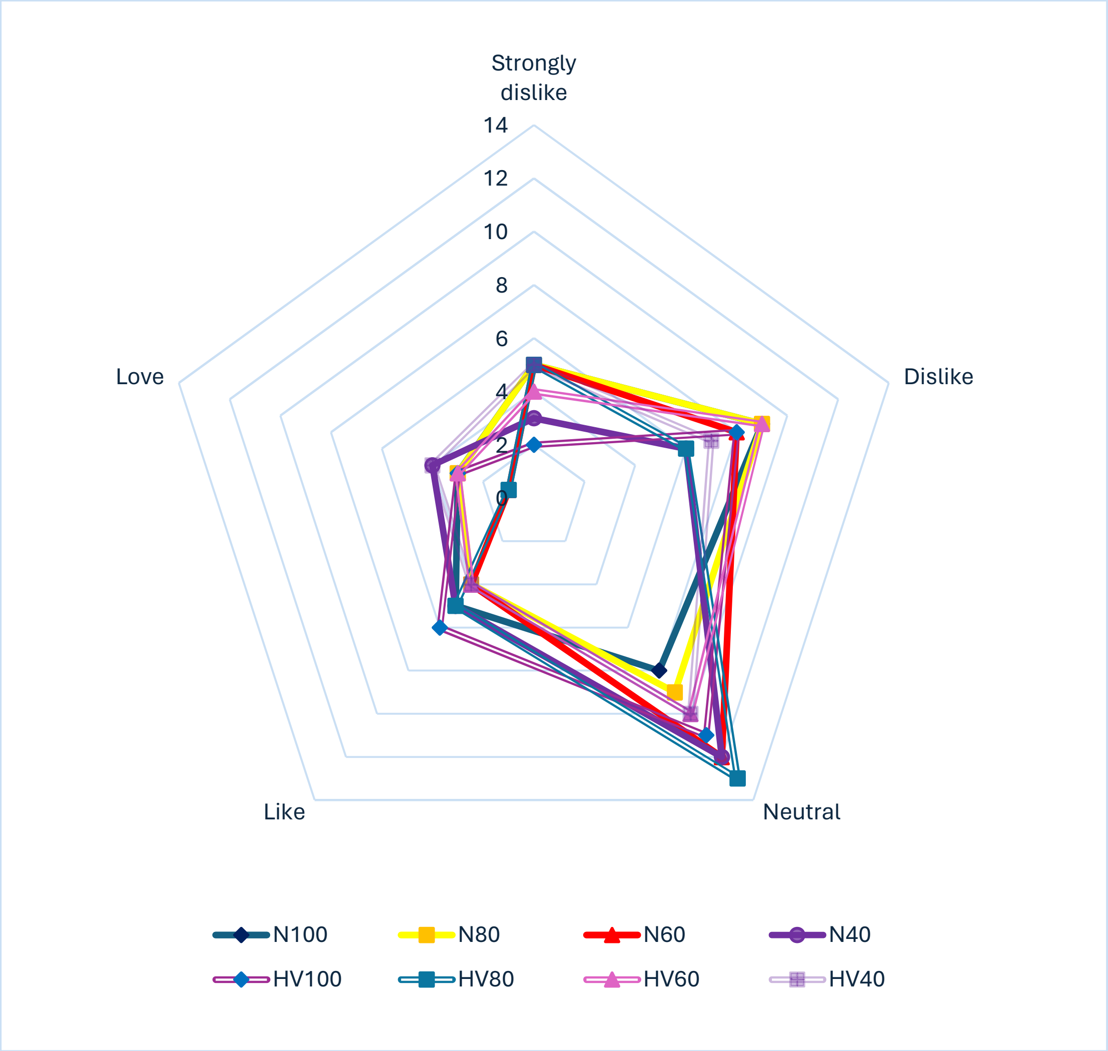

Figure S3. Results of the organoleptic analysis of parameter Smell in the fermented drink.
